# P13 Complex outpatient antimicrobial therapy (CoPAT) activity data for the Acute Care at Home (ACAH) in Cornwall, UK

**DOI:** 10.1093/jacamr/dlaf239.017

**Published:** 2026-01-05

**Authors:** Daniel Hearsey, Nicola Leigh, Sarah Crowle, Jennie Stephens

**Affiliations:** Pharmacy Department, Royal Cornwall Hospital NHS Foundation Trust, Cornwall, UK; Pharmacy Department, Royal Cornwall Hospital NHS Foundation Trust, Cornwall, UK; Cornwall Partnership NHS Foundation Trust, Cornwall, UK; Intensive Care Department, Royal Cornwall Hospital NHS Foundation Trust, Cornwall, UK

## Abstract

**Background:**

The Acute Care at Home (ACAH) team delivers secondary care-level treatment to prevent admission and facilitate early discharge for patients across Cornwall with infectious and non-infectious conditions in their own homes. The service operates jointly between Cornwall Partnership NHS Foundation Trust (CPFT) and Royal Cornwall Hospital NHS Foundation Trust (RCHT); it operates in-line with the BSAC OPAT Good practice recommendations guidelines.^1^ It is led by a clinician from RCHT (Consultant in Acute and Intensive Care Medicine) and supported by an Antimicrobial Pharmacist from RCHT and a Consultant Microbiologist from RCHT; clinical assessment, IV and oral therapies are delivered by the team of specialist nurses employed by CPFT.

**Objectives:**

To quantify annual CoPAT activity, focusing on total treatment days delivered; additional treatment days missed due to delayed or declined referrals caused by capacity constraints was also collected as a secondary outcome.

**Methods:**

All patients receiving care under the ACAH are recorded on the electronic handover sheet which is updated daily by the duty nurse. This sheet was reviewed daily on weekdays by the Antimicrobial Stewardship (AMS) Pharmacist or AMS Pharmacy Technician, and the following baseline information was recorded for every patient on a monthly Excel sheet: name, NHS number, referral source, date treatment with ACAH commenced and location within Cornwall. The treatment delivered to the patient was also entered onto this data collection sheet, which would include: IV antibiotics (IVA), oral antibiotics (PO), IV diuretics (IVD), IV fluids (IVF), IV blood products (IVB), blood monitoring only (BO) and any other IV drugs, excluding antibiotics, diuretics, fluids and blood (IVO). This information was collected for June 2024 – May 2025. To quantify the missed activity due to deferred or declined referrals, the patient’s details were recorded by the duty nurse on receipt of a referral where the referral was unable to commence due to lack of capacity within the service. The Consultant Lead for the service reviewed every electronic referral made to the service and identified all delayed acceptance.

**Results:**

Between June 2024 to May 2025, the ACAH team delivered a total of 10 338 treatment days; 10 039 (97%) were for the management of infectious diagnoses, and 299 (3%) for non-infectious diagnoses. Of the infection management cohort, 6259 days (63%) were nurse-delivered IV therapy, 516 days (5%) were patient self-administered IV therapy, 3015 days (30%) of complex oral antibiotic regimes and 213 (2%) of monitoring only. Capacity limitations resulted in 2098 missed treatment days, representing a potential 20% increase in service delivery.

**Conclusions:**

ACAH provides safe, effective home-based care across Cornwall’s challenging geography, supporting NHS aims to manage more patients at home.^2^ Despite delivering over 10 000 therapy days, capacity constraints limit further expansion. Future audits incorporating patient complexity will be key to evaluating and developing this evolving service.

## References

[1] Chapman ALN et al Updated good practice recommendations for outpatient parenteral antimicrobial therapy (OPAT) in adults and children in the UK. JAC Antimicrob Resist 2019; 1: dlz026. 10.1093/jacamr/dlz026 PMC8209972 34222901

[2] Department of Health and Social Care . Fit for the Future: 10 Year Health Plan for England – Executive Summary. 2025. https://www.gov.uk/government/publications/10-year-health-plan-for-england-fit-for-the-future/fit-for-the-future-10-year-health-plan-for-england-executive-summary

